# Anesthesia Usage and Pain Management in Colposcopy: A Scoping Review of Efficacy and Approaches

**DOI:** 10.7759/cureus.70384

**Published:** 2024-09-28

**Authors:** Miis Akel, Dhruv Ratra, Maggie Wright, Crystal Barroca, Amy A Abdou, Paul Kaldas, Shreya Bhatt, Aleymi Perez, Sahil Shah, Sergio Hernandez Borges

**Affiliations:** 1 Obstetrics and Gynecology, Nova Southeastern University Dr. Kiran C. Patel College of Osteopathic Medicine, Clearwater, USA; 2 Anesthesiology, Nova Southeastern University Dr. Kiran C. Patel College of Osteopathic Medicine, Miami, USA; 3 Integrative Medicine, Nova Southeastern University Dr. Kiran C. Patel College of Osteopathic Medicine, Miami, USA; 4 Cardiology, Nova Southeastern University Dr. Kiran C. Patel College of Osteopathic Medicine, Clearwater, USA; 5 Physical Medicine and Rehabilitation, Nova Southeastern University Dr. Kiran C. Patel College of Osteopathic Medicine, Boca Raton, USA; 6 Physical Medicine and Rehabilitation, Nova Southeastern University Dr. Kiran C. Patel College of Osteopathic Medicine, Fort Lauderdale, USA; 7 Ophthalmology, Nova Southeastern University Dr. Kiran C. Patel College of Osteopathic Medical School, Fort Lauderdale, USA; 8 Obstetrics and Gynecology, Lake Erie College of Osteopathic Medicine, Bradenton, USA; 9 Physical Medicine and Rehabilitation, Larkin Community Hospital, Palm Springs Campus, Miami, USA; 10 Internal Medicine/Family Medicine, Larkin Community Hospital, Palm Springs Campus, Miami, USA

**Keywords:** cancer cervical, cervical assessment, cervical biopsy, colposcopy, general obgyn, local anesthetic, pap-smear, topical anesthetic, type of anesthesia, lidocaine

## Abstract

Colposcopies are diagnostic procedures conducted to detect precancerous and cancerous lesions on the cervix and are often done as a sequelae of histological abnormalities found on cervical screening exams, such as precancerous abnormalities, positive human papillomavirus (HPV) results, or a past medical history of cervical dysplasia. Colposcopies, while often necessary to ensure the health of the patient, can induce a lot of anxiety and distress in the time leading up to the procedure, often due to fear of the procedure itself, anticipation of pain, as well as fear regarding the results of the colposcopy. Experiencing physical pain and discomfort during gynecological procedures can negatively influence a woman's willingness to comply with future medical appointments, often exacerbating feelings of anxiety and distress. A systematic search was conducted to identify the relevant articles published within the last 10 years pertaining to pain management in colposcopy procedures. The search strategy aimed to identify studies involving the utilization of anesthesia or analgesics for pain management or prevention during colposcopy, with a focus on randomized controlled trials. This systematic review aims to review the existing literature on the use of anesthetics in colposcopy and its effect on patient outcomes, investigating the current strategies of anesthesia use in the setting of a colposcopy, as well as their efficacy in improving pain.

## Introduction and background

Colposcopies are diagnostic procedures used to detect precancerous and cancerous lesions on the cervix, ensuring early detection and treatment. These procedures are typically performed in a gynecological setting and are commonly conducted as follow-ups to evaluate abnormalities detected during cervical screening exams, such as Papanicolaou (Pap) smears [1]. During a colposcopy, the patient is positioned similarly to a pelvic exam, and a colposcope (a specialized magnifying device) is used to closely examine the cervix, vagina, and vulva for abnormal tissue. Acetic acid or Lugol’s iodine may be applied to the cervix to highlight abnormal areas for biopsy. Biopsies of suspicious areas may then be conducted for histopathological analysis.

Colposcopies are indicated when there is an immediate risk of cervical intraepithelial neoplasia (CIN) 2 or higher, abnormal Pap smear findings, or abnormal cytology and histology results [2]. They are also performed on women with underlying risks for cervical precancer, such as positive high-risk human papilloma virus (HPV) testing, atypical cervical tissue, and a past medical history of cervical dysplasia [3].

According to the guidelines from the American College of Obstetricians and Gynecologists, women over the age of 21 should be screened for cervical cancer via cytology collected from a Pap smear every three years until the age of 65. For those aged 30 to 65, co-testing with both a Pap smear and HPV testing every five years is also recommended as an alternative screening method [4]. Patients who have had a hysterectomy with cervix removal should follow these guidelines if they have a history of high-grade cervical precancerous lesion or cervical cancer [4]. Women with atypical squamous cells of undetermined significance (ASC-US) under the age of 25 can either repeat their Pap test in one year or opt for HPV testing. If HPV negative, they can resume their regular cervical cancer screening schedule; if HPV positive, they should repeat their Pap test in one year [4]. Women over the age of 25 may either repeat their Pap testing in one year, or preferably undergo HPV testing [4]. If HPV positive, except if their last HPV test within the past five years was negative, they are recommended to undergo a colposcopy [4]. Women with high-grade squamous intraepithelial lesions (ASC-H), regardless of age, must get a colposcopy to rule out cervical cancer [5]. Notable contraindications for colposcopy include active cervical or vaginal infections and certain colposcopy procedures during pregnancy, such as the endocervical curettage (ECC) [6].

When the criteria for conducting colposcopies are met, the procedure can be utilized to obtain biopsies from regions displaying potentially premalignant or malignant characteristics. Proper illumination and magnification are crucial to visualize cervical lesions, which are identified based on changes in color, contour, and vascular patterns after applying 3%-5% acetic acid to the cervical region [7]. To ensure comprehensive diagnostic coverage, biopsies should be taken from two to four distinct sites, focusing on the transition zone of the cervix where the squamocolumnar junction is located. While the steps taken during colposcopies can cause discomfort, they enable effective treatment upon the detection of preinvasive disease [7].

Colposcopies are essential for the maintenance of women’s health; however, due to their invasive nature, many women experience severe discomfort, leading to non-compliance. Inconsistent provision of adequate anesthesia during colposcopies results in many women experiencing negative effects, including pain and discomfort associated with the procedure. Various parts of the colposcopy exam can elicit pain, such as the initial insertion or prolonged use of the speculum, colposcopy-directed punch biopsies [8], and the ECC when indicated. Research has shown that ECC is often one of the most painful aspects of a colposcopy [9].

Several factors can affect the level of pain experienced during the procedure. For example, tenaculum replacement has been associated with increased pain [8]. Additionally, postmenopausal patients with genitourinary syndrome of menopause may find the speculum insertion to be more painful and harder to tolerate [10]. The literature indicates that pain experienced during a colposcopy often leads to psychological consequences [11]. Specifically, some women experience significant distress before undergoing colposcopies due to apprehension about the procedure, its potential complications, the expectation of pain, and the fear of having cancer [12]. After the procedure, there is evidence of post-colposcopy anxiety, with self-reported pain being the strongest predictor [13]. The pain endured during a colposcopy can negatively impact a patient’s compliance in returning for recommended follow-up treatments and may deter women from seeking adequate cervical screenings in the future [13].

Despite the availability of anesthetic options, their use during colposcopy remains uncommon in current practice. Local or general anesthesia can be administered depending on patient preference or the complexity of the colposcopically directed treatment [14]. Local anesthesia, such as lidocaine injections, is often favored as it requires fewer resources, shortens recovery time, and facilitates a faster procedure overall [15]. General anesthesia, while available for patients with high levels of anxiety, poor access to cervical lesions, or based on patient preference, is less commonly used due to its higher morbidity risks and resource demands [15]. The underuse of anesthesia during colposcopy may be attributed to perceptions that the procedure is tolerable without it, along with concerns about resource allocation and the potential risks of more invasive anesthesia [16]. However, studies indicate that even with thorough physician-patient communication about the procedure, patients frequently experience anxiety and distress, highlighting a potential gap in patient-centered care [17]. Addressing this underutilization could improve patient comfort and overall experience during colposcopy.

While colposcopies are essential for the early detection and treatment of cervical abnormalities, the associated discomfort and psychological distress can significantly impact women's broader approach to healthcare. Anxiety and discomfort during the procedure often lead to non-compliance, avoidance of follow-up care, and apprehension toward future gynecological exams. Addressing these concerns through better pain management, including the appropriate use of anesthesia, is critical for improving patient comfort and fostering trust in preventive care. This scoping review examines the current methods of anesthesia used during colposcopy with the aim of enhancing the patient experience. By emphasizing the need for further research into pain management strategies, this review focuses on optimizing patient care in gynecological procedures to improve healthcare outcomes and encourage patient adherence.

## Review

Methods

A systematic search was conducted on PubMed on March 22, 2024, to identify relevant articles published within the last 10 years (2014-2024) pertaining to pain management in colposcopy procedures. The search strategy aimed to identify studies involving the utilization of anesthesia or analgesics for pain management or prevention during colposcopy, with a focus on randomized controlled trials. The search strategy utilized the following keywords and Medical Subject Headings (MeSH) terms: "colposcopy," "pain management," "anesthesia," "analgesics," and variations thereof. Filters were applied to restrict the search to articles published in the English language.

Exclusion criteria were applied to filter out irrelevant studies and to maintain the focus on original research specific to anesthesia usage in colposcopy procedures. Reviews, including meta-analyses, literature reviews, and scoping reviews, were excluded to ensure that only primary studies reporting novel data were considered. Case reports were also excluded due to their anecdotal nature, and studies focusing on loop electrosurgical excision procedures (LEEP) were omitted because LEEP typically involves general anesthesia in operating rooms in the United States, which falls outside the scope of this review. To ensure a rigorous selection process and minimize bias, a total of eight reviewers were involved in the article screening using Rayyan software. Following the initial search, duplicate articles were removed. The remaining articles were independently screened based on titles and abstracts according to the inclusion and exclusion criteria. Discrepancies between reviewers were discussed, and consensus was reached through group discussions. Full-text articles were then assessed for eligibility.

**Figure 1:**
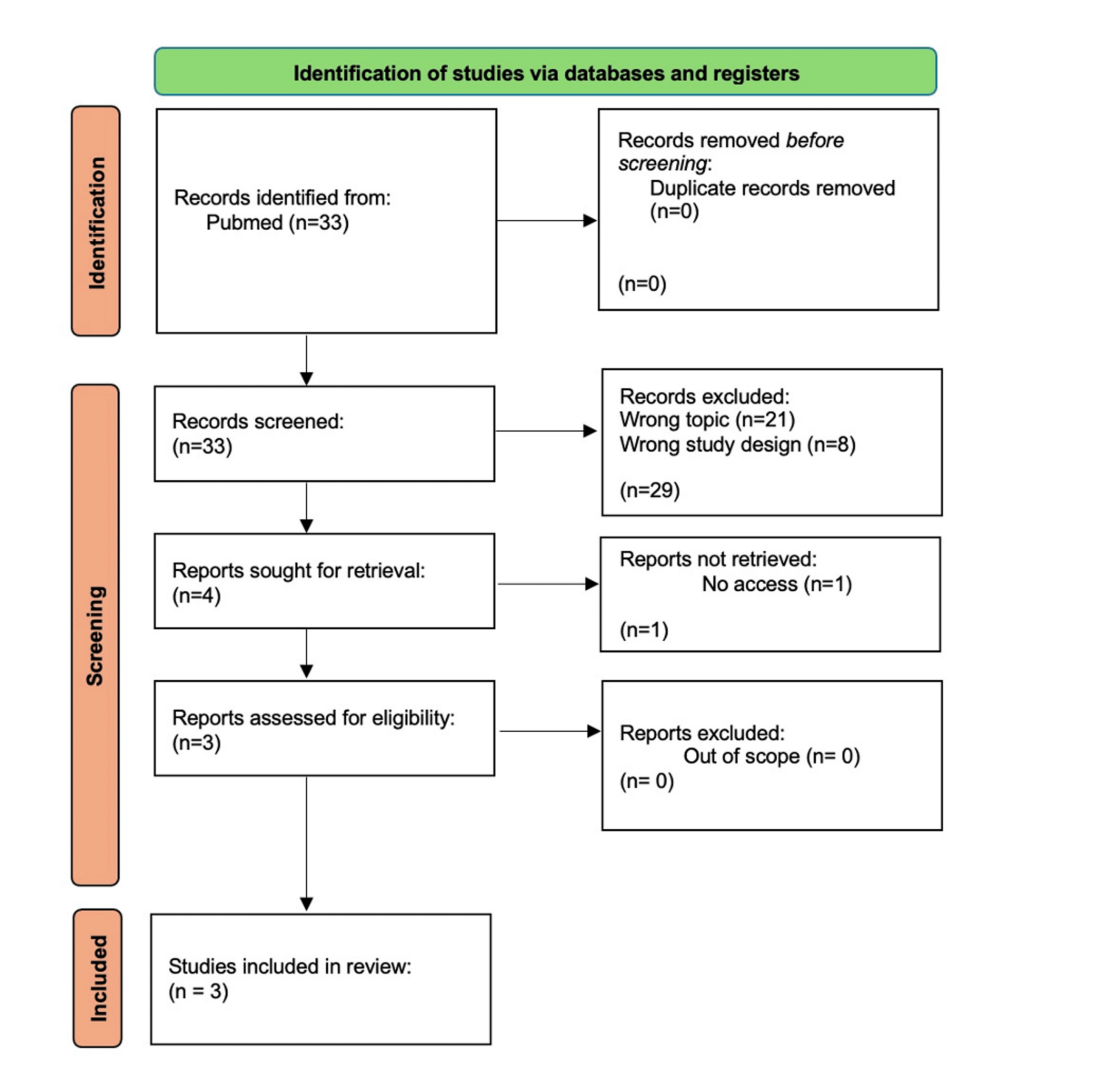
PRISMA diagram outlining inclusion and exclusion criteria for article selection. PRISMA: Preferred Reporting Items for Systematic Reviews and Meta-Analyses

The final inclusion criteria comprised studies conducted between 2014 and 2024, involving colposcopy procedures performed as outpatient services, and reporting the use of anesthesia or analgesics for pain management or prevention. Articles that did not meet these criteria were excluded. A Preferred Reporting Items for Systematic Reviews and Meta-Analyses (PRISMA) flow diagram was used to illustrate the article selection process, as shown in Figure 1.

The initial search yielded 33 articles. Of these, eight articles were excluded based on publication type, one article was excluded due to full-text ineligibility, and 21 articles were excluded due to their focus on LEEP procedures. This process resulted in a final selection of three articles that met the inclusion criteria for further analysis.

Results

There are various methods for managing pain in colposcopy procedures. Three studies investigated the efficacy of different methods as outlined in Table 1, including the application of lidocaine topical 10% spray [18,19] or the administration of local anesthetics such as a combination of prilocaine and felypressin injection [20] during the procedure. Notably, all studies accounted for confounding variables such as age, parity, dysmenorrhea, menopause, and history of LEEP without observing alterations in outcomes [18-20].

**Table 1:** Summary of studies included in this review. CDB: colposcopy-directed cervical biopsy VAS: Visual Analog Scale

Reference	Study Design	Data Collection	Study Aim	Findings	Recommendations	Limitations
Wongluecha T et al. (2017) [18]	Prospective randomized controlled trial	Women with an abnormal cervical screening test requiring colposcopy (n=200) from April to December 2015. Assigned to two groups: one received lidocaine spray, the other no anesthesia. A 10-point Likert scale was used at different stages of the procedure.	To investigate the efficacy of topical 10% lidocaine spray in reducing pain during colposcopy-directed cervical biopsy (CDB) compared to no anesthesia.	There were no significant differences in baseline characteristics between the lidocaine spray and no anesthesia groups. Pain scores at baseline, biopsy, and post-procedure were comparable between the two groups. However, the mean difference in pain scores from baseline to biopsy and post-procedure was statistically higher in the no-anesthesia group.	Lidocaine spray did not significantly reduce pain during CDB compared to no anesthesia. The pain associated with the procedure was generally minimal, indicating that additional interventions to reduce pain may be unnecessary.	The study did not employ a placebo in the control group, which could have influenced outcomes. Complete blinding of participants and colposcopists was not achievable due to the nature of the procedure.
Öz et al. (2015) [19]	Randomized, placebo-controlled, double-blind trial	A total of 214 women randomly assigned; 104 in the lidocaine group and 110 in the control group.	To compare the effectiveness of topical lidocaine spray compared to a placebo during colposcopy cervical biopsies and endocervical curettage.	Pain scores after cervical biopsy were similar in both groups. Mean standard deviation pain scores associated with cervical biopsy were 2.18 + 1.7 in the lidocaine group, and 2.13 + 1.6 in the control group.	No evidence to recommend the routine use of routine locally sprayed lidocaine anesthesia before cervical punch biopsy or endocervical curettage.	A major limitation of the study was the lack of a placebo control group. Patient receiving lidocaine injection were aware that they received “the medicine” creating a major bias in the pain perception of the individuals.
Kiviharju et al. (2017) [20]	Prospective randomized cohort study	Women undergoing colposcopies and punch biopsies (n=198) from November 2012 and September 2013. 99 women were in the control (no anesthesia) and 99 women were in the study group (local anesthesia). Used visual analog scale (VAS) for pain scores and adjusted for potential confounders.	To evaluate the efficacy of local anesthesia during colposcopies and punch biopsies compared to no anesthesia.	Pain scores for injection of local anesthetic were significantly lower than for cervical punch biopsies without anesthesia. Adjusted analyses showed statistically significant differences in pain scores between the groups during all procedure categories. Women receiving local anesthesia had significantly lower probabilities of experiencing unbearable pain compared to those without anesthesia.	Local anesthesia during colposcopies and punch biopsies effectively reduces pain perception. Incorporating local anesthesia into these procedures can enhance patient comfort and compliance.	The subjective nature of VAS scores, potential selection bias due to patient preferences for anesthesia, and the lack of blinding. Additionally, while statistically significant, the clinical significance of the observed pain score differences may vary. Further research could explore alternative pain management methods and their impact on patient outcomes.

Among the reviewed studies, two found no significant differences in pain levels between patients who received topical lidocaine spray before the colposcopy and those who underwent the procedure without any anesthesia [18.19]. However, Wong's study reported statistically significant findings regarding biopsy-related pain scores. Although there was no statistically significant difference between the control group (no anesthesia) and the experimental group (topical lidocaine spray) for pain experienced during the biopsy itself, there were significant differences in pain scores between baseline and the procedure, as well as between post-procedure and baseline [18]. In this context, 'baseline' refers to the initial pain level reported before the procedure started, serving as a reference point for comparison throughout the procedure. Specifically, the mean difference in pain from baseline to the procedure, and from baseline to post-procedure, was higher in patients who did not receive any anesthesia [18]. This suggests that the application of topical lidocaine, compared to no anesthesia, enhanced overall patient comfort and pain management. Interestingly, although initial discomfort during speculum insertion was slightly higher in the lidocaine group, these patients experienced less pain during the biopsy itself. Additionally, post-procedural pain was reduced, indicating an overall improvement in comfort. While differences in pain during the biopsy were not statistically significant, the cumulative effect of reduced insertion and post-procedure pain demonstrated a meaningful improvement in patient comfort.

Furthermore, Kiviharju et al.'s study investigated the variance in pain scores between procedures conducted without anesthesia and those administered with locally injected anesthetics [20]. Utilizing a Visual Analog Scale (VAS) to quantify pain, the study revealed significantly higher VAS pain values in the groups without anesthesia compared to those with injected local analgesia, even after adjusting for low pain thresholds in certain individuals [20]. Moreover, the likelihood of experiencing "unbearable pain" was halved in patients who received anesthesia compared to those who did not during cervical biopsy [20]. Several factors influence the efficacy of anesthesia, including individual pain thresholds and education level [19-20]. This suggests that personal pain thresholds play a significant role in modifying the effects of local anesthesia during the procedure [20]. Additionally, Öz et al.’s study identified an inverse relationship between pain levels and education level suggesting that higher education levels correlate with lower pain values [19].

Discussion

This review explores the efficacy of lidocaine spray and lidocaine injection as pain management strategies for colposcopy procedures. While some studies found no significant difference in pain levels between patients who received lidocaine topical spray and those without anesthesia, Wongluecha et al. revealed noteworthy disparities in biopsy-related pain scores [18]. Specifically, patients treated with lidocaine spray reported improved comfort and greater pain management compared to those without anesthesia, suggesting that there are possible advantages to lidocaine topical spray in colposcopy procedures. Although the differences in pain during biopsies were not statistically significant, the cumulative effect of insertion and post-procedure pain levels demonstrated a significant improvement [18]. Similarly, other studies have highlighted the role of local anesthetics in ameliorating pain during gynecologic procedures by alleviating discomfort from speculum and cervical insertions. For instance, Aksoy's study demonstrated significant pain reduction during intrauterine device (IUD) insertion using 10% lidocaine spray alone [21]. Further research should explore how anesthesia can alleviate different aspects of the procedure, including speculum insertion, biopsy, speculum removal, and post-procedural discomfort.

The administration of local anesthesia varies, including painless lidocaine spray or more invasive analgesic injection, each with varying degrees of efficacy. However, Oyama et al. suggest a reduction in pain scores for cervical biopsies, endocervical curettage, and overall procedure scores with lidocaine injection, highlighting its effectiveness in decreasing perceived pain during colposcopy [22]. Additionally, Karasu et al. demonstrated the effectiveness of lidocaine spray in reducing pain during IUD insertion, emphasizing its ease of application and rapid onset of action [23]. Conversely, while paracervical lidocaine injection improved pain levels during IUD insertions, it had no effect on tenaculum-related pain [23]. This study also suggested that analgesic injections have painful administration that makes them less preferable. Conversely, Kiviharju et al. indicated that the injection itself induced less discomfort than the biopsy, suggesting that the pain experienced during injection might be more tolerable compared to other aspects of the procedure [20]. Throughout the various studies [18-23], a central emphasis was placed on understanding and improving patient experience during colposcopy procedures. The three studies included in this review explore the themes of patient-reported pain levels, comfort throughout the procedure, and whether local anesthesia can aid in improving the patient experience. In examining these factors, the studies sought to identify strategies for mitigating pain and enhancing overall comfort.

Despite the insights gained, methodological limitations persist across the studies, including the lack of blinding, potential bias due to the absence of a placebo control group, and the subjective nature of pain assessment scales. These limitations, coupled with the limited number of studies investigating anesthesia for colposcopy, suggest potential for bias due to selection bias. Moreover, the absence of blinding and placebo control groups in some studies further compounds the methodological limitations, leading to potentially biased results. Placebo control groups are crucial for future research to enhance the validity of the results. Additionally, the subjective nature of pain assessments, influenced by individual pain interpretation and tolerance, underestimates the need for careful consideration in interpreting study findings. One notable study by Wong et al. showed a significant difference in pain levels between local anesthesia patients and the placebo group during speculum insertion and post-procedure stages, but not during the actual steps of the procedure [18].

Current evidence on the use of anesthetics during colposcopy shows mixed results. While some studies, such as Wong's, indicate that topical lidocaine may provide modest improvements in patient comfort, particularly post-procedure, others report no significant differences in pain levels during the biopsy itself compared to procedures without anesthesia. These findings suggest that while anesthetics can enhance patient comfort in certain aspects of the procedure, their overall impact on pain reduction and patient outcomes may be limited or variable depending on the specific anesthetic method and patient population studied. Additionally, due to the visceral innervation of the uterine cavity, local anesthesia procedures may be ineffective in providing pain relief. While current guidelines recommend oral analgesics prior to the procedure, their efficacy remains uncertain [5]. Therefore, more importantly, it is recommended to properly explain the procedure to patients and mentally prepare them for the biopsy. Moreover, considering the risks and rewards of anesthetics is essential when dealing with these procedures. In general, minimal pain was associated with the procedures studied, and additional interventions may carry risks of allergy or infection which may outweigh the insignificant pain difference. Further research is needed to ascertain if applying anesthesia at specific stages of the procedure would provide relief, particularly during speculum insertion, acetic acid application, and endocervical colposcopy or biopsy. Additionally, variations in timing and technique yield different results, as demonstrated by Wong's study administering the spray prior to the biopsy step, which decreased the level of pain difference [18], leading to the areas of further study. Furthermore, research on specific demographics requiring additional pain relief, such as chronic pelvic pain, dysmenorrhea, and high pain sensitivity, is warranted. Also, it is important to monitor for factors such as socioeconomic background which may confound pain scores. Conclusively, for the common patient, additional anesthetic may be redundant in its effects [19], but there is definitely room for further study in perfecting the art of colposcopy.

## Conclusions

Anesthesia may provide some benefits in pain management during colposcopy, however routine use for all patients may not be warranted. This review examined the effectiveness of lidocaine spray and injection in managing pain during colposcopy procedures, highlighting some of the benefits of utilizing local anesthesia during different stages of the procedure. Additionally, further research is needed to explore the timing and technique of anesthesia administration, as well as its effectiveness in specific demographic groups with high pain sensitivity. Overall, an individualized approach, adequate patient education, and further research are necessary to optimize pain management strategies and patient experience during colposcopy procedures.
